# Secondary Hemophagocytic Lymphohistiocytosis Triggered by Visceral Leishmaniasis in a Patient With Multiple Sclerosis Receiving Fingolimod Therapy: A Diagnostic Challenge

**DOI:** 10.7759/cureus.119391

**Published:** 2026-11-09

**Authors:** Karolina Wypasek, Daniel Lüscher, Matthias von Kietzell, Thomas Lehmann

**Affiliations:** 1 Internal Medicine, Endocrinology and Diabetology, Cantonal Hospital St. Gallen, HOCH Health Ostschweiz, St. Gallen, CHE; 2 Cardiology, Cantonal Hospital St. Gallen, HOCH Health Ostschweiz, St. Gallen, CHE; 3 Infectious Diseases, Infektiologie Praxis St. Gallen, St. Gallen, CHE; 4 Hematology and Medical Oncology, Cantonal Hospital St. Gallen, HOCH Health Ostschweiz, St. Gallen, CHE

**Keywords:** fever of unknown origin, fingolimod, hemophagocytic lymphohistiocytosis, hlh, multiple sclerosis, opportunistic infection, visceral leishmaniasis

## Abstract

Hemophagocytic lymphohistiocytosis (HLH) is a rare but life-threatening hyperinflammatory syndrome characterized by uncontrolled activation of macrophages and lymphocytes, resulting in a cytokine storm, multiorgan dysfunction, and substantial mortality despite appropriate treatment. In adults, HLH is most commonly secondary to infections, malignancies, or autoimmune disorders. Less common but increasingly recognized triggers include drug-induced and iatrogenic causes, as well as severe cutaneous adverse reactions such as drug reaction with eosinophilia and systemic symptoms (DRESS). The spectrum of infectious triggers varies with geographic exposure, and visceral leishmaniasis, a recognized but uncommon trigger of secondary HLH, may therefore present a considerable diagnostic challenge in non-endemic regions, particularly in immunocompromised patients.

We present the case of a 68-year-old man with relapsing-remitting multiple sclerosis who was receiving fingolimod and presented with persistent high-grade fever, progressive weakness, bicytopenia, transaminitis, and splenomegaly. Despite extensive microbiological, serological, radiological, and histopathological investigations, the underlying cause remained elusive. The clinical, histopathological, and laboratory findings, including extreme hyperferritinemia, hypertriglyceridemia, hypofibrinogenemia, and cytopenias, fulfilled 6 of 8 HLH-2004 diagnostic criteria, yielding an HScore of 292 and a probability of greater than 99% for reactive HLH. Immunosuppressive therapy was initiated because of impending organ failure. Splenectomy, indicated by splenic necrosis, provided the diagnostic specimen. Ultimately, polymerase chain reaction (PCR) testing of splenic tissue and bone marrow established the diagnosis of visceral leishmaniasis. Targeted treatment with liposomal amphotericin B resulted in sustained clinical recovery, with no evidence of recurrence over 7.5 years of follow-up.

This case highlights the importance of considering HLH in patients with fever of unknown origin and cytopenias, particularly when receiving immunomodulatory therapy. Furthermore, it illustrates the diagnostic challenges associated with visceral leishmaniasis in non-endemic regions and raises the possibility that fingolimod-induced immune modulation increases the risk of opportunistic infections and disease reactivation.

## Introduction

Hemophagocytic lymphohistiocytosis (HLH) is a severe hyperinflammatory syndrome caused by uncontrolled activation of cytotoxic T lymphocytes, natural killer cells, and macrophages, resulting in excessive cytokine production and potentially fatal multiorgan dysfunction [1-5].

Although HLH was initially described in pediatric populations, growing awareness has led to increased recognition of adult-onset HLH, which is most frequently associated with infections, malignancies, and autoimmune disorders [1,2,5,6]. In addition to these, drug-induced and iatrogenic forms are increasingly recognized, and HLH may occur in association with severe cutaneous adverse reactions, in particular drug reaction with eosinophilia and systemic symptoms (DRESS), resulting in overlapping clinical and laboratory features [3,4].

Diagnosing HLH in adults is challenging because its symptoms overlap with those of sepsis, severe infections, hematologic malignancies, and autoimmune diseases. Current diagnostic approaches rely predominantly on the HLH-2004 criteria, as described by Henter et al. [7], and the HScore developed by Fardet et al. [8], both of which are widely used diagnostic tools in adults [1]. Early initiation of treatment is crucial because mortality remains high even with adequate therapy [1-6].

Visceral leishmaniasis is a parasitic disease caused by intracellular protozoa of the genus *Leishmania*. The disease primarily affects the reticuloendothelial system and can present with prolonged fever, hepatosplenomegaly, pancytopenia, and hyperferritinemia. Secondary HLH is a recognized complication of visceral leishmaniasis [9,10].

Immunosuppressive therapies, including sphingosine-1-phosphate receptor modulators such as fingolimod, may increase susceptibility to opportunistic infections and facilitate reactivation of latent pathogens [11-15].

We present a case of visceral leishmaniasis-associated HLH in a patient receiving fingolimod for multiple sclerosis, highlighting the associated diagnostic challenges.

This report was prepared in accordance with the CARE (Case Report) guidelines for clinical case reporting [16].

## Case presentation

A 68-year-old man receiving fingolimod therapy for multiple sclerosis presented to our tertiary care hospital with persistent fever, chills, headache, and progressive weakness. He denied experiencing cough, dyspnea, gastrointestinal symptoms, or urinary complaints.

A review of his medical history revealed a diagnosis of relapsing-remitting multiple sclerosis. One year prior to admission, disease-modifying therapy had been switched to fingolimod due to the presence of disease activity. Despite progressive pyramidal symptoms, the patient demonstrated independence in activities of daily living and reported a satisfactory quality of life.

His travel history was notable for a several-year stay in Ghana until 2000, during which he contracted malaria; a three-week stay in Ghana four months before presentation; and travel to Costa Rica two years before presentation.

Upon admission, the patient’s body temperature was 39.5 °C. He appeared ill but was hemodynamically stable and fully oriented. The physical examination was unremarkable, with the exception of pyrexia. No lymphadenopathy, skin lesions, or signs of meningism were identified.

The initial laboratory evaluation revealed an elevated C-reactive protein level of 58 mg/L (reference range <8 mg/L); bicytopenia with normocytic anemia (hemoglobin 109 g/L, reference range 140-180 g/L; mean corpuscular volume (MCV) 88 fL, reference range 80-95 fL) and leukopenia (leukocyte count 2.0 × 10⁹/L, reference range 4.0-10.0 × 10⁹/L); mild hyponatremia of 128 mmol/L (reference range 136-144 mmol/L); and elevated liver enzymes, with AST levels of 141 U/L and ALT levels of 101 U/L (reference range AST <40 U/L, ALT <55 U/L). Urinalysis was unremarkable. Chest radiography showed no infiltrates. Cranial computed tomography and cerebrospinal fluid analysis were normal.

In the absence of an identifiable infectious source and considering the patient’s immunocompromised status, empirical treatment with ceftriaxone was initiated, and fingolimod therapy was discontinued.

A subsequent abdominal computed tomography scan revealed splenomegaly accompanied by multiple heterogeneous splenic lesions, initially raising suspicion of splenic abscesses (Figure 1). Repeated blood cultures remained sterile. Despite comprehensive diagnostic evaluations and empirical antimicrobial therapy, the patient exhibited recurrent fevers up to 40 °C and progressive clinical deterioration.

**Figure 1:**
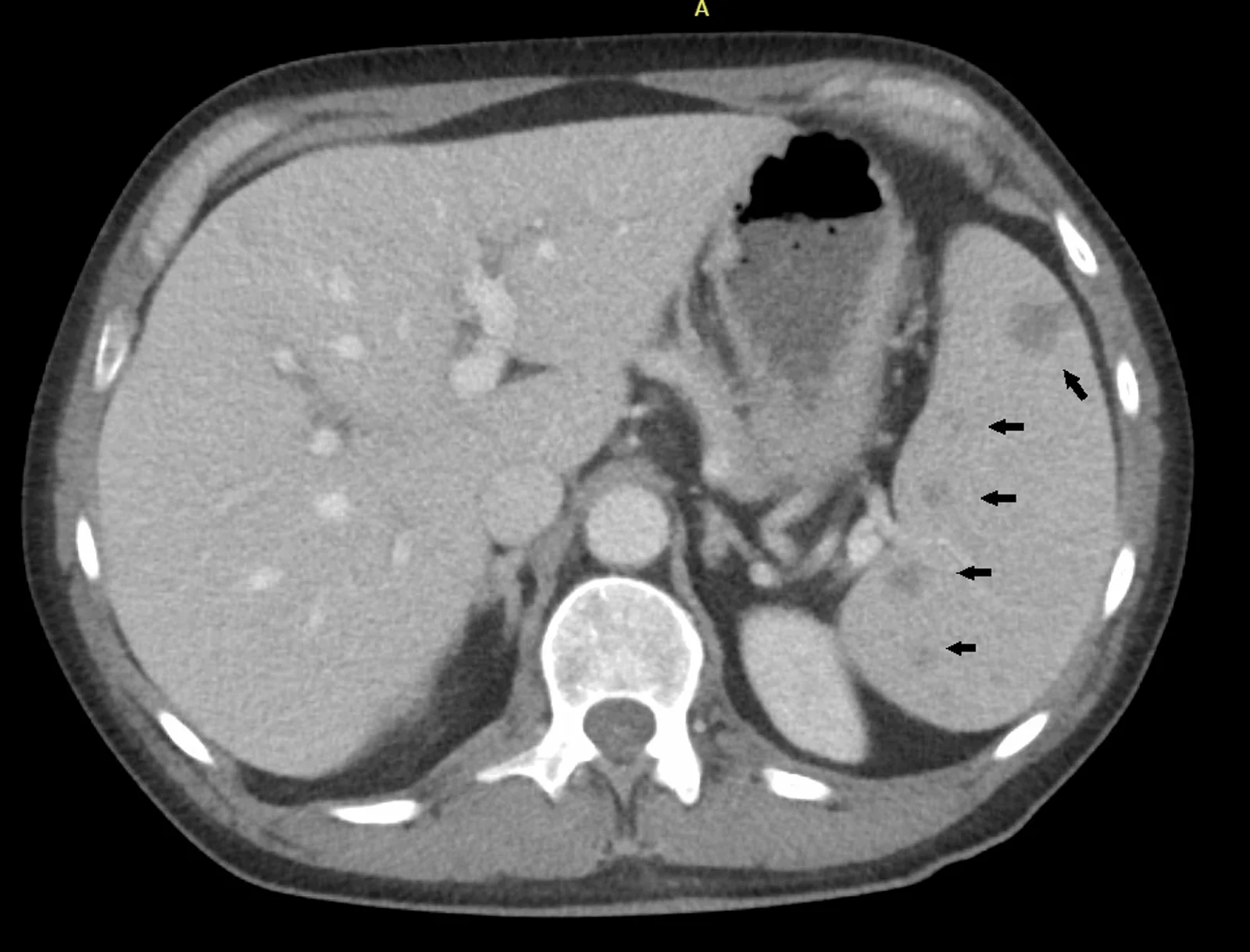
Contrast-enhanced abdominal computed tomography demonstrating marked splenomegaly with multiple heterogeneous hypodense splenic lesions (arrows), initially considered suspicious for splenic abscesses. Subsequent PCR analysis of splenic tissue confirmed visceral leishmaniasis.

A comprehensive infectious workup was performed. Serological testing included *Coxiella burnetii*, *Brucella* species, *Bartonella henselae*, *Toxoplasma gondii*, *Rickettsia *species, *Francisella tularensis*, HIV, hepatitis C virus, and herpes simplex virus. Molecular testing included hepatitis E virus RNA, cytomegalovirus DNA, Epstein-Barr virus DNA, and influenza A and B virus RNA. *Cryptococcus neoformans* antigen testing and repeated malaria smears and antigen tests were also performed. These investigations did not identify an infectious cause. Notably, *Leishmania* serology showed a mildly elevated IgG titer, prompting further molecular investigation. Differential diagnoses included occult hematologic malignancy, particularly lymphoma, disseminated fungal infections, tuberculosis, and other opportunistic infections associated with immunomodulatory therapy.

Percutaneous biopsy of the splenic lesions was complicated by post-interventional hemorrhage with hemorrhagic shock, requiring angiographic embolization and one night of intensive care. Histopathological examination of the specimen was non-diagnostic, showing necrotic tissue without evidence of active infection or lymphoma. Three days later, acute abdominal pain prompted repeat computed tomography, which demonstrated splenic necrosis with intraparenchymal gas.

Because of persistent clinical deterioration and ongoing fever without an established diagnosis, empirical corticosteroid therapy with oral prednisone 140 mg daily (2 mg/kg) was initiated and continued for five days. Liver biopsy and bone marrow aspiration were performed the following day. The liver biopsy showed portal inflammation with lobular necrosis corresponding to the splenic changes, but no pathogen was identified on special stains and no lymphoma was found.

^18^F-Fluorodeoxyglucose positron emission tomography/computed tomography (^18^F-FDG PET/CT) revealed diffuse splenic uptake without evidence of lymphoma or another focal infectious source (Figure 2).

**Figure 2:**
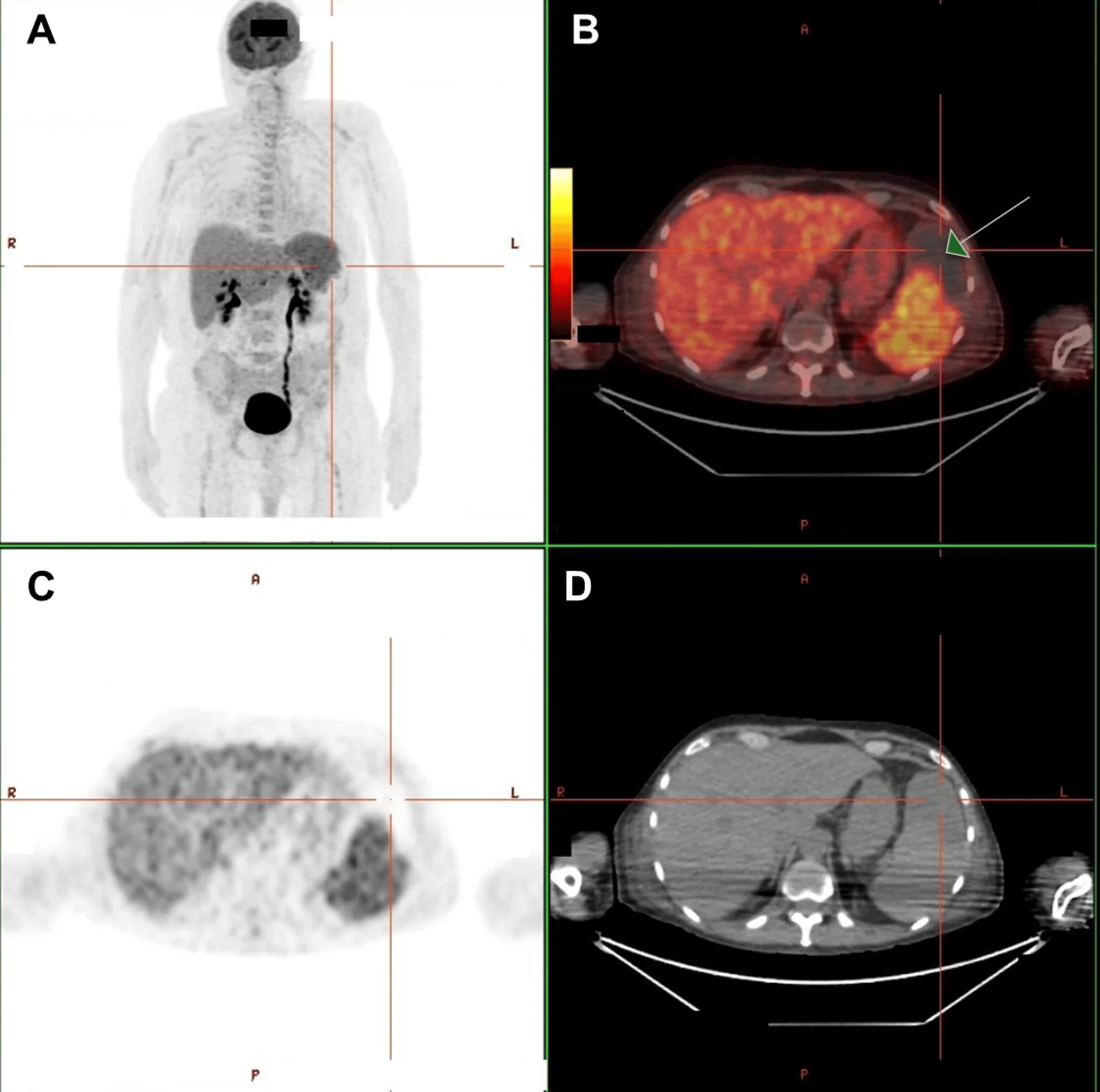
18F-FDG PET/CT findings (A) Coronal maximum-intensity projection (MIP) image showing splenomegaly and diffusely increased FDG uptake in the hematopoietic bone marrow of the axial skeleton. (B) Axial fused PET/CT image at the level of the spleen demonstrating splenomegaly with homogeneously increased FDG uptake (maximum SUV 5.8) and photopenia (arrow) corresponding to the splenic infarction documented on the preceding CT. (C) Corresponding axial PET image at the same level showing splenic FDG uptake. (D) Corresponding axial low-dose CT image at the same level demonstrating splenic enlargement. No focal malignant lesion, hypermetabolic lymphadenopathy, or alternative infectious source was identified. FDG, fluorodeoxyglucose; MIP, maximum intensity projection; SUV, standardized uptake value

Bone marrow examination showed marked hypercellularity with trilineage hematopoiesis, dysplastic erythropoiesis, and hemophagocytic features, without evidence of hematologic malignancy (Figure 3).

**Figure 3:**
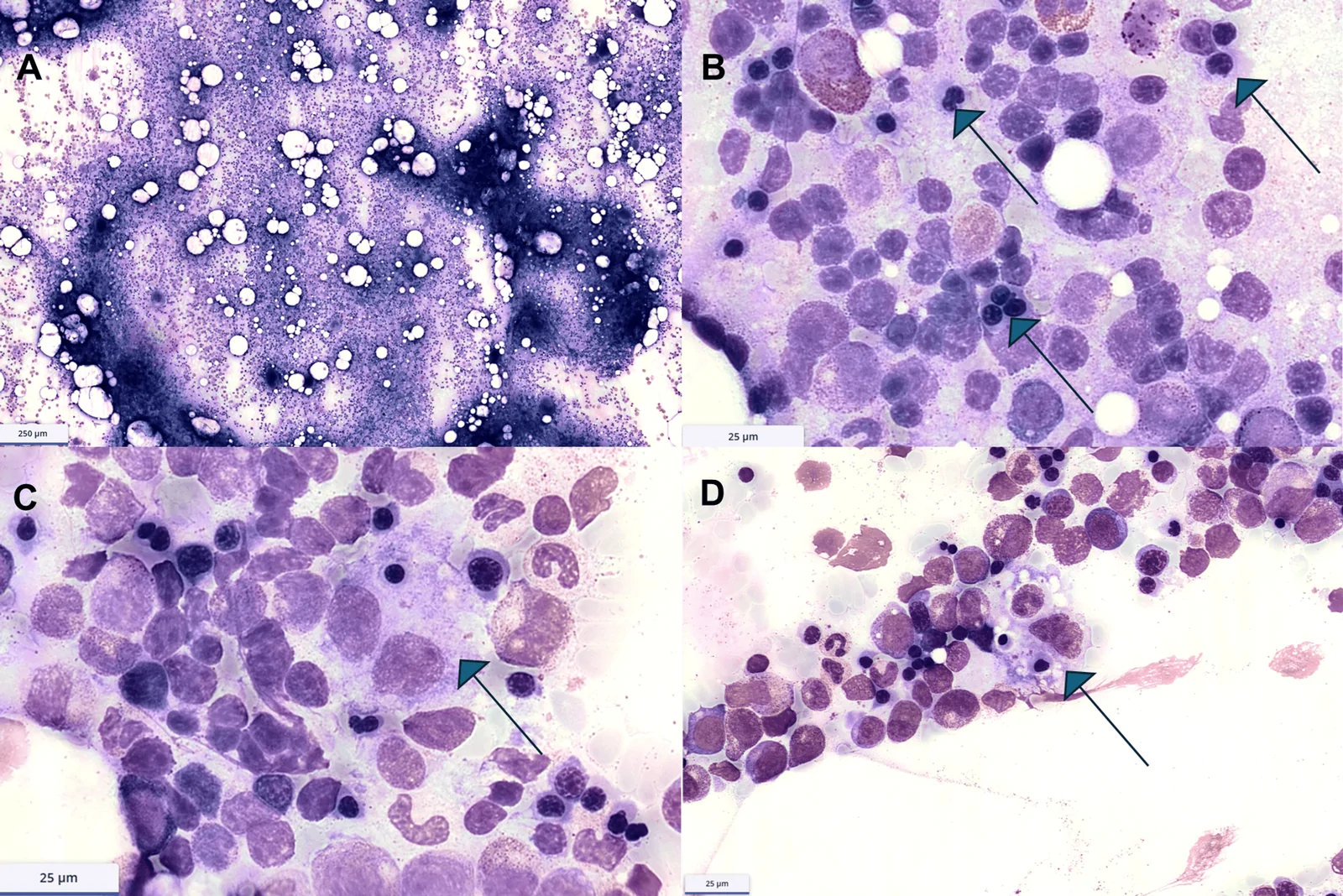
Bone marrow aspirate findings A) Low-power overview showing hypercellularity with preserved fat spaces (scale bar: 250 µm). (B) Dysplastic erythroblasts (arrows). (C, D) Macrophages containing phagocytosed hematopoietic cells (arrows), consistent with hemophagocytosis. Scale bars in panels B–D: 25 µm. May-Grünwald-Giemsa stain.

These findings, together with the laboratory results, raised a strong suspicion of hemophagocytic lymphohistiocytosis (HLH). Ferritin rose from an initial level of 13,730 µg/L to 103,992 µg/L (reference range 30-330 µg/L). The patient’s clinical presentation, which included hypertriglyceridemia (3.4 mmol/L; reference range <1.7 mmol/L), hypofibrinogenemia (0.77 g/L; reference range 1.5-3.5 g/L), worsening bicytopenia (the nadir value was 68 g/L for hemoglobin; the leukocyte and granulocyte counts reached nadirs of 1.1 × 10⁹/L and 0.8 × 10⁹/L, respectively, while the platelet count, although mildly reduced (nadir 134 × 10⁹/L), remained above the thresholds defined in the HLH-2004 criteria and the HScore), persistent fever, splenomegaly, and hemophagocytic features in the bone marrow strongly supported this diagnosis. The patient fulfilled 6 of 8 HLH-2004 criteria (Table 1), and the calculated HScore was 292 (Table 2). This corresponds to a probability greater than 99% of reactive hemophagocytic syndrome [8].

**Table 1:** HLH-2004 diagnostic criteria fulfilled by the patient Total criteria fulfilled: 6 of 8; HLH-2004 diagnostic criteria according to Henter et al. [7]. HLH, hemophagocytic lymphohistiocytosis; NK, natural killer

HLH-2004 Criterion	Patient Value	Criterion Met
Fever ≥38.5 °C	Maximum temperature 39.5 °C	Yes
Splenomegaly	Splenomegaly confirmed by abdominal CT	Yes
Cytopenias affecting ≥2 cell lines	Two of three lines met: hemoglobin nadir 68 g/L (threshold <90 g/L); granulocyte nadir 0.8 × 10⁹/L (threshold <1.0 × 10⁹/L); platelet nadir 134 × 10⁹/L (threshold <100 × 10⁹/L, not met)	Yes
Hypertriglyceridemia and/or hypofibrinogenemia	Triglycerides 3.4 mmol/L; fibrinogen 0.77 g/L	Yes
Hemophagocytosis in bone marrow, spleen, or lymph nodes	Hemophagocytic features observed in bone marrow	Yes
Ferritin ≥500 µg/L	Ferritin 103,992 µg/L	Yes
Low or absent NK-cell activity	Not assessed	—
Elevated soluble CD25 (sIL-2 receptor)	Not assessed	—

**Table 2:** HScore calculation ^a^Fingolimod was scored as underlying immunosuppression (18 points). If not classified as such, the HScore is 274 instead of 292; both correspond to a probability greater than 99% of reactive hemophagocytic syndrome. ^b^Cytopenia thresholds in the HScore (original units): hemoglobin ≤9.2 g/dL, leukocyte count ≤5,000/mm³, and/or platelet count ≤110,000/mm³. ^c^The initial ferritin level was 13,730 µg/L; the peak value was used for scoring. HScore criteria adapted from Fardet et al. [8]. AST, aspartate aminotransferase.

Variable	Patient value	HScore scoring criteria	Score
Known underlying immunosuppression^a^	Fingolimod therapy	No: 0 points; Yes: 18 points	18
Temperature	39.5 °C	<38.4 °C: 0; 38.4–39.4 °C: 33; >39.4 °C: 49	49
Organomegaly	Splenomegaly	None: 0; hepatomegaly or splenomegaly: 23; hepatomegaly and splenomegaly: 38	23
Cytopenias^b^	Bicytopenia	1 lineage: 0; 2 lineages: 24; 3 lineages: 34	24
Ferritin^c^	103,992 µg/L (reference range: 30–330 µg/L)	<2,000 µg/L: 0; 2,000–6,000 µg/L: 35; >6,000 µg/L: 50	50
Triglycerides	3.4 mmol/L (reference range: <1.7 mmol/L)	<1.5 mmol/L: 0; 1.5–4.0 mmol/L: 44; >4.0 mmol/L: 64	44
Fibrinogen	0.77 g/L (reference range: 1.5–3.5 g/L)	>2.5 g/L: 0; ≤2.5 g/L: 30	30
AST	141 U/L (reference range: <40 U/L)	<30 U/L: 0; ≥30 U/L: 19	19
Hemophagocytosis on bone marrow aspirate	Present	No: 0; Yes: 35	35
Total HScore			292

In light of impending hepatic failure and life-threatening hyperinflammation, treatment was escalated to intravenous methylprednisolone 1 g daily for six days, followed by 250 mg daily for five days. Intravenous immunoglobulin 30 g daily was administered concomitantly for five days (cumulative dose 150 g, 2.1 g/kg). With the underlying trigger still unidentified and given the necrotic spleen, splenectomy was performed the following day, both as a therapeutic measure and to obtain diagnostic tissue.

Because HLH activity persisted, therapy was intensified according to the HLH-94/2004 protocol with four doses of etoposide 150 mg/m², a repeat methylprednisolone pulse with subsequent stepwise tapering, conversion to dexamethasone 10 mg/m² daily, and cyclosporine 150 mg twice daily (target trough 100-150 µg/L). Trimethoprim-sulfamethoxazole and valacyclovir prophylaxis were given throughout (Table 3).

**Table 3:** Clinical timeline. Day 1 corresponds to the day of admission AST, aspartate aminotransferase; CRP, C-reactive protein; CSF, cerebrospinal fluid; CT, computed tomography; HLH, hemophagocytic lymphohistiocytosis; MRI, magnetic resonance imaging; PCR, polymerase chain reaction; ^18^F-FDG PET/CT, ^18^F-fluorodeoxyglucose positron emission tomography/computed tomography

Day	Clinical course and investigations	Treatment
1	Admission with fever 39.5 °C; CRP 58 mg/L, hemoglobin 109 g/L, leukocytes 2.0 × 10⁹/L, AST 141 U/L; blood cultures, urinalysis, chest radiograph and influenza swab negative	Fingolimod discontinued
2	Persistent fever and headache; cranial CT and CSF analysis normal	Ceftriaxone 2 g/day
3	Rising transaminases; ultrasound and CT showing splenomegaly with multiple hypodense splenic lesions suspicious for abscesses; granulocyte nadir 0.8 × 10⁹/L	—
4	Echocardiography without signs of endocarditis and cranial MRI without septic emboli	Ceftriaxone discontinued
5	Percutaneous splenic biopsy complicated by hemorrhage with hemorrhagic shock; angiographic embolization; one night of intensive care; daily fever up to 40 °C	—
7	Further rise in liver enzymes; ultrasound consistent with reactive hepatitis	—
8	Acute abdominal pain; CT showing splenic necrosis with intraparenchymal gas	—
9	Persistent fever without diagnosis; all blood cultures negative	Prednisone 140 mg/day (2 mg/kg)
10	Liver biopsy and bone marrow aspiration; splenic histology non-diagnostic	—
13	^18^F-FDG PET/CT without lymphoma or infectious focus; bone marrow compatible with hemophagocytosis; ferritin 103,992 µg/L; HLH diagnosed (6/8 criteria, HScore 292)	Methylprednisolone 1 g/day (days 13–18); intravenous immunoglobulin 30 g/day (days 14–18); aciclovir
14	Splenectomy	—
15–17	Defervescence and marked clinical improvement; approximately tenfold reduction in ferritin within three days.	—
19–23	—	Methylprednisolone reduced to 250 mg/day
22–24	*Campylobacter *enteritis and paralytic subileus	Azithromycin; conservative management
24	Persisting HLH activity	Etoposide 150 mg/m² (dose 1 of 4); methylprednisolone re-escalated to 1 g/day (days 24–25)
25	—	Cyclosporine 150 mg twice daily
26	—	Etoposide (dose 2); methylprednisolone 500 mg/day (days 26–28)
27	Fascial dehiscence requiring surgical repair	—
29–33	Severe hypoalbuminemia (14.7 g/L)	Methylprednisolone 100 mg/day; enteral nutrition
32	—	Etoposide (dose 3)
34	—	Dexamethasone 10 mg/m²/day
35	*Leishmania *PCR from splenic tissue positive; species-specific PCR identifying *L. donovani*; bone marrow PCR confirmatory	Liposomal amphotericin B 300 mg (5 mg/kg), dose 1 of 9; etoposide (dose 4)
40–42	Sustained recovery	Cyclosporine, dexamethasone and antimicrobial prophylaxis tapered and discontinued
46	Transfer to inpatient rehabilitation; recovery continued	Liposomal amphotericin B continued during rehabilitation, reaching a cumulative dose of 2,700 mg (45 mg/kg)

The patient improved markedly following splenectomy and intensification of immunosuppressive therapy. Ferritin concentrations decreased approximately tenfold within three days.

The initial histopathological evaluation of the spleen remained inconclusive. However, because of a mildly elevated *Leishmania* IgG titer, PCR testing was performed on splenic tissue, which confirmed the presence of *Leishmania* DNA. Species-specific PCR identified *Leishmania donovani*, and subsequent molecular testing of bone marrow specimens also confirmed visceral leishmaniasis.

Consequently, targeted therapy with liposomal amphotericin B was initiated at 300 mg intravenously (5 mg/kg) for nine doses over one month, resulting in a cumulative dose of 2,700 mg (approximately 45 mg/kg). HLH-directed immunosuppression was tapered from day 5 onward, and cyclosporine, dexamethasone, and antimicrobial prophylaxis were discontinued seven days after the first dose of liposomal amphotericin B. The postoperative course was complicated by *Campylobacter* enteritis, paralytic ileus, a pancreatic fistula, fascial dehiscence, and severe hypoalbuminemia requiring enteral nutrition, from which the patient recovered steadily.

The patient was transferred to inpatient rehabilitation on day 46 and completed the full training program, during which hematologic parameters, ferritin, and liver enzymes normalized. Approximately four years later, the patient received cyclosporine and prednisolone for about one year for an unrelated inflammatory skin condition, without reactivation of leishmaniasis or recurrence of HLH.

Fingolimod was not resumed, and no alternative disease-modifying therapy for multiple sclerosis was initiated. His multiple sclerosis progressed during long-term follow-up. At 7.5 years of follow-up, he remains free of clinical evidence of leishmaniasis or HLH and is receiving no immunosuppressive therapy.

## Discussion

Adult HLH remains underrecognized because its clinical manifestations frequently mimic those of severe infection, sepsis, or hematologic malignancy. Diagnostic delay is associated with adverse outcomes and an increased mortality rate [1-6]. Current diagnostic approaches rely predominantly on the HLH-2004 criteria, as described by Henter et al. [7], and the HScore developed by Fardet et al. [8], both of which are widely used in adults [1]. Although originally developed for pediatric patients, the HLH-2004 criteria are widely applied in adults [1,7,8]. The patient satisfied six out of eight criteria (five of which are required for diagnosis). The HScore indicated a greater than 99% probability of HLH.

The prognosis of secondary HLH depends strongly on the underlying etiology. In a nationwide German cohort of adult patients, overall mortality was 51%, with 20.6% of deaths occurring within 30 days of diagnosis. Median survival differed substantially by trigger: 160 days in malignancy-associated HLH, 248 days in HLH of unknown cause, 641 days in infection-associated HLH, and not reached in macrophage activation syndrome, although this difference did not reach statistical significance in that cohort [17]. Our patient's course was consistent with the relatively favourable prognosis of infection-associated HLH.

Infections account for a substantial proportion of secondary HLH in adults, reported in 39% of cases in a single-center analysis [18] and in 44.5% of a nationwide German registry cohort, in which infection even exceeded malignancy as the most frequent trigger [17]. Epstein-Barr virus remains the single most frequent infectious agent, followed by cytomegalovirus and mycobacterial infection [17,18]. Because a broad search for infectious triggers is therefore warranted in every patient, the diagnosis of HLH should not be regarded as an endpoint but as the starting point for an intensified investigation. Visceral leishmaniasis is a well-established infectious trigger of secondary HLH [9,10]. Given that *Leishmania* parasites proliferate within macrophages of the reticuloendothelial system, patients may present with fever, splenomegaly, cytopenias, hyperferritinemia, and laboratory abnormalities that closely resemble HLH. Consequently, distinguishing primary manifestations of visceral leishmaniasis from HLH may be difficult because of substantial clinical overlap [9,10].

The spectrum of infectious triggers is strongly influenced by geographic exposure. Whereas cytomegalovirus and mycobacterial infections predominate in European cohorts, other pathogens are relevant in different regions and should be considered according to the individual travel history [17,18]. In our patient, the exposure history pointed towards West Africa, where visceral leishmaniasis is documented but uncommon [19,20].

Isolated reports have described the association between fingolimod therapy and opportunistic infections, including visceral leishmaniasis [14]. Other reports have described HLH in patients with visceral leishmaniasis, underscoring the need to consider this diagnosis in patients with protracted fever and cytopenias, particularly in those with a history of travel to endemic regions [9,10]. In our patient, extensive serological, histopathological, and imaging investigations were initially inconclusive. The diagnosis was ultimately established by species-specific polymerase chain reaction (PCR) targeting the *Leishmania *mini-exon locus with subsequent sequencing, performed at a reference laboratory for parasitology, which confirmed *Leishmania donovani* in splenic tissue and bone marrow. This finding illustrates the value of molecular diagnostics in immunocompromised patients. Immunocompromised patients may fail to mount a detectable antibody response, and *Leishmania* serology is unreliable in this setting and cannot distinguish acute infection from past exposure. Direct detection of *Leishmania* DNA by PCR in bone marrow, spleen, or other affected tissue is the method of choice in immunocompromised patients, in whom serology may raise suspicion but a negative result does not exclude visceral leishmaniasis.

A detailed exposure history is essential for guiding diagnostic testing toward plausible pathogens. However, in patients with progressive organ dysfunction, and particularly in life-threatening disease, the workup must remain deliberately broad, combining serology with pathogen-specific PCR on all available tissue, even when this entails parallel and costly testing.

Fingolimod sequesters lymphocytes within lymph nodes through the modulation of sphingosine-1-phosphate receptors, altering immune surveillance and potentially increasing susceptibility to opportunistic infections [11-14]. Individual cases of visceral leishmaniasis and of infection-associated HLH have been reported separately in fingolimod-treated patients (Table 4). To the best of our knowledge, this represents the first reported case of visceral leishmaniasis-associated HLH occurring during fingolimod therapy.

**Table 4:** Published individual case reports of visceral leishmaniasis and/or hemophagocytic lymphohistiocytosis in fingolimod-treated patients with multiple sclerosis EBV, Epstein-Barr virus; HSV-2, herpes simplex virus type 2; *L.*, *Leishmania*; VL, visceral leishmaniasis; M, male; F, female

Author	Year	Visceral leishmaniasis	HLH	Comment	Age/Sex	Outcome
Abreu et al. (Conference abstract) [12]	2014	No	Yes	EBV-associated HLH under fingolimod	39/M	Died (day 31)
Artemiadis et al. (Case report) [11]	2015	Yes	No	*L. infantum* infection under fingolimod	37/F	Full recovery
Ikumi et al. (Case report) [13]	2016	No	Yes	HSV-2-associated HLH under fingolimod	56/M	Died (day 18)
Guerra-Amor et al. (Case report) [14]	2022	Yes	No	Leishmaniasis under fingolimod	33/M	Recovery, no relapse at 6 months
Present case	2026	Yes	Yes	VL-associated HLH under fingolimod	68/M	Recovery, no relapse at 7.5 years

Drug-induced HLH, including the DRESS/HLH overlap syndrome, represents an important differential diagnosis in patients receiving new or recently changed medications, as its clinical and laboratory features overlap substantially with those of infection-triggered HLH [3,4]. Both conditions typically present with fever, rash, and eosinophilia, and delayed recognition is associated with higher mortality. In our patient, neither rash nor eosinophilia was present, and the diagnosis was established by direct pathogen detection, making a drug-induced etiology unlikely.

Two mechanisms could explain the timing of infection in our patient. The three-week stay in Ghana four months before presentation is consistent with primary infection, as the incubation period of visceral leishmaniasis typically ranges from two to six months. Alternatively, the infection may have been acquired decades earlier during his extended residence in Ghana [19,20], with asymptomatic persistence and subsequent reactivation under fingolimod-associated impairment of immune surveillance. Although visceral leishmaniasis is uncommon and incompletely characterized in West Africa, asymptomatic infection may persist for years, and progression to overt visceral disease depends on the interaction between parasite factors and host immunity [15], so both mechanisms remain plausible [11,14,15]. Notably, Birndt et al. identified three cases of visceral leishmaniasis-associated HLH in a nationwide German registry, confirming that this trigger is encountered in non-endemic settings when exposure history is carefully obtained [17].

Another important aspect of this case is the role of splenectomy. While splenectomy is not a standard intervention in the management of HLH [1], it was primarily indicated in our patient by splenic necrosis, provided the diagnostic specimen, and was followed by clinical improvement. The rapid decline in ferritin following surgery raises the possibility that removal of the markedly involved spleen contributed to reducing the inflammatory burden, while also enabling definitive diagnosis.

Splenic infarction was considered as an alternative explanation for the splenic lesions in this case. For colleagues outside Europe, tick-borne pathogens, such as *Babesia microti,* warrant consideration as a possible cause of splenic infarction where the exposure history is compatible. In our case, however, the splenic necrosis was documented three days after percutaneous biopsy, which had been complicated by hemorrhage requiring angiographic embolization. This temporal relationship suggests that the necrosis was related to the intervention rather than to an independent infarction.

Current recommendations for adult HLH emphasize rapid control of hyperinflammation while simultaneously identifying and treating the underlying trigger [1,5,6]. In this particular case, the initiation of HLH-directed therapy prior to the identification of the causative pathogen was deemed appropriate, given the progressive organ dysfunction and the high mortality rate associated with untreated HLH. Following confirmation of visceral leishmaniasis, liposomal amphotericin B therapy addressed the underlying infection, thereby enabling discontinuation of HLH-directed immunosuppression. Relapse of visceral leishmaniasis is well described in immunocompromised hosts, and secondary prophylaxis is often considered in this setting. The absence of clinical relapse despite a subsequent year-long course of cyclosporine and corticosteroids supports a durable clinical response, although parasitological cure cannot be verified by any currently available test, and relapse surveillance therefore relies on clinical assessment, which is the accepted approach in this setting.

This report has several limitations. As a single case, it cannot establish a causal relationship between fingolimod therapy and visceral leishmaniasis-associated HLH. The precise timing and mechanism of infection remain uncertain, as both recent acquisition and reactivation of a latent infection are compatible with the exposure history. Furthermore, no parasitological testing was available from the earlier period. Soluble CD25 levels and NK-cell activity were not measured; nevertheless, the patient fulfilled six of eight HLH-2004 diagnostic criteria without these parameters.

Despite these limitations, several clinically relevant lessons emerge from this case. First, HLH should be considered in the differential diagnosis of fever of unknown origin accompanied by cytopenias and hyperferritinemia [1-6]. Second, exposure history should guide but never restrict the diagnostic workup, particularly in patients receiving immunomodulatory therapies [1-6]. Third, molecular testing of tissue specimens can establish the diagnosis when conventional investigations remain inconclusive, as illustrated by a case in which *Leishmania*-specific PCR was diagnostic despite negative serology and a non-diagnostic bone marrow aspirate [10]. In our patient, the serological finding was only mildly elevated and did not permit a definitive diagnosis without molecular confirmation. Molecular testing is particularly relevant in immunocompromised patients, in whom serological testing can be less reliable. Finally, when progressive organ dysfunction threatens survival, HLH-directed therapy should be initiated before the underlying trigger has been identified [1,5,6].

## Conclusions

Secondary HLH should be considered in patients with prolonged fever, cytopenias, splenomegaly, and extreme hyperferritinemia, particularly when conventional infectious investigations are unrevealing. Visceral leishmaniasis is an important infectious trigger of HLH and may be difficult to diagnose when the relevant exposure history is not initially apparent. Molecular testing of tissue specimens can be valuable when conventional investigations are inconclusive.

In patients receiving fingolimod or other immunomodulatory therapies, opportunistic infections should remain part of the differential diagnosis. Early recognition and treatment of both HLH and its underlying trigger are essential. In our patient, targeted antiparasitic therapy was followed by sustained clinical remission without recurrence of leishmaniasis or HLH over 7.5 years.
